# A FRAMEWORK FOR CARE TRANSITIONS FOR OLDER ADULTS WITH COMPLEX HEALTH CONDITIONS

**DOI:** 10.1093/geroni/igz038.2534

**Published:** 2019-11-08

**Authors:** Paul Stolee, Jacobi B Elliott, Kerry Byrne, Joanie Sims-Gould, Catherine Tong, Bert Chesworth, Mary Egan, Dorothy Forbes

**Affiliations:** 1 University of Waterloo, Waterloo, Ontario, Canada; 2 University of British Columbia, Vancouver, British Columbia, Canada; 3 Western Health Sciences, London, Ontario, Canada; 4 University of Ottawa, Ottawa, Ontario, Canada; 5 University of Alberta, Edmonton, Alberta, Canada

## Abstract

For older adults with complex health conditions, transitions between care settings are common and a major risk to quality of care and patient safety. Care transition interventions have shown positive impacts on continuity of care and health service use, however, most require additional human resources (e.g., transition coach), focus on one transition or “handoff”, and provide support for individual patients without addressing underlying challenges of health system integration. We sought to develop a framework for system-level enhancements to care transitions for older adults. We report a secondary framework analysis of an ethnographic investigation (the “InfoRehab” project) of care transitions for older persons who had experienced a hip fracture. The ethnographic study involved interviews, observations, and document reviews for 23 patients, 19 family caregivers, and 92 health care providers. Data were collected at each transition point (1-4/patient) along the care continuum, at three Canadian sites (large urban, mid-size urban, rural). Our framework analysis followed the approach described by Gale et al. (2013), using as cases 12 peer-reviewed papers which had reported InfoRehab results. Two researchers coded findings from each paper, then developed an analytical framework of eight themes by consensus; these include: patient involvement and choice, family caregiver involvement, patient complexity, health care provider coordination, information sharing, documentation, system constraints, and relationships. NVivo 11 was used to index findings into these themes and to generate a matrix. We are working with system stakeholders, including patients and caregivers, to apply this framework in the development of improved systems for care transitions.

